# Large pinning forces and matching effects in YBa_2_Cu_3_O_7-*δ*_ thin films with Ba_2_Y(Nb/Ta)O_6_ nano-precipitates

**DOI:** 10.1038/srep21188

**Published:** 2016-02-18

**Authors:** Lars Opherden, Max Sieger, Patrick Pahlke, Ruben Hühne, Ludwig Schultz, Alexander Meledin, Gustaaf Van Tendeloo, Rainer Nast, Bernhard Holzapfel, Marco Bianchetti, Judith L. MacManus-Driscoll, Jens Hänisch

**Affiliations:** 1 IFW Dresden, Institute for Metallic Materials, P.O. Box 270116, 01171 Dresden, Germany; 2 TU Dresden, Institute for Solid-State Physics, 01062 Dresden, Germany; 3 Dresden High Magnetic Field Laboratory (HLD-EMFL), Helmholtz-Zentrum Dresden-Rossendorf, 01328 Dresden, Germany; 4 University of Antwerp, EMAT Research Group, Groenenborgerlaan 171, 2020 Antwerp, Belgium; 5 KIT, Institute for Technical Physics, Hermann-von-Helmholtz-Platz 1, 76344 Eggenstein-Leopoldshafen,Germany; 6 University of Cambridge, Department of Materials Science and Metallurgy, 27 Charles Babbage Rd., Cambridge, CB3 0FS, UK

## Abstract

The addition of mixed double perovskite Ba_2_Y(Nb/Ta)O_6_ (BYNTO) to YBa_2_Cu_3_O_7−*δ*_ (YBCO) thin films leads to a large improvement of the in-field current carrying capability. For low deposition rates, BYNTO grows as well-oriented, densely distributed nanocolumns. We achieved a pinning force density of 25 GN/m^3^ at 77 K at a matching field of 2.3 T, which is among the highest values reported for YBCO. The anisotropy of the critical current density shows a complex behavior whereby additional maxima are developed at field dependent angles. This is caused by a matching effect of the magnetic fields *c*-axis component. The exponent *N* of the current-voltage characteristics (inversely proportional to the creep rate *S*) allows the depinning mechanism to be determined. It changes from a double-kink excitation below the matching field to pinning-potential-determined creep above it.

YBa_2_Cu_3_O_7−*δ*_ (YBCO) based coated conductors have large potential in such diverse applications as wires/cables, motors/generators, high-field coils, and superconducting permanent magnets, each of them with a certain range of temperature and magnetic field and a certain need in magnitude and isotropicity in critical current density *J*_*c*_ . In order to use the full potential of YBCO, it is mandatory to tailor its transport properties for the envisaged application. It is therefore necessary to understand how the critical current density *J*_c_ behaves over wide ranges of magnetic field strength *H*, temperature *T* and angle *θ* between *H* and the crystallographic *c*-axis. The *J*_c_(*H*,*T*,*θ*) dependence, being determined by the underlying pinning landscape in a complex way^1^, can be raised and adjusted by the creation of artificial defects within the superconductor which act as pinning centers. This can be achieved by e.g. irradiation^2^, substrate decoration^3^,^4^, rare-earth substitution^5^,^6^, and incorporation of secondary phases. The latter one is relatively inexpensive and easy and therefore commonly used. After initial studies on naturally growing nanoparticles in YBCO thin films, such as Y_2_O_3_^7^ (recently investigated again more closely as artificial pinning centers^8^,^9^), first investigations on artificial nanoparticles were made on BaZrO_3_^10^. *J*_*c*_ could be strongly improved through incorporating this or related barium perovskites Ba*M*O_3_ (*M* transition metal) because they grow as globular or columnar structures which act as pinning centers. This has been reported for various techniques such as pulsed laser deposition (PLD)^11^,^12^,^13^,^14^,^15^ or metal-organic chemical vapor deposition (MOCVD)^16^. In chemical deposition methods (CSD, MOD), usually randomly oriented, more or less isotropic nanoparticles are formed^17^,^18^,^19^. Secondary phases can furthermore introduce concurrent defects in the YBCO matrix. Strong positive correlations between nanostrain and *J*_*c*_ for example suggest that nanostrain and strain-induced defects such as stacking faults^20^ or dislocations^21^ act as pinning centers. Nanoinclusions of the double perovskites Ba_2_YNbO_6_ (BYNO) and Ba_2_YTaO_6_ (BYTO) have been suggested^22^ as promising pinning centers due to several advantages compared to BZO. Nb or Ta ions are less likely to substitute for Y in YBCO, which should lead to a smaller *T*_*c*_ reduction^22^, (a minute Nb substitution on Cu was even reported to slightly increase *T*_*c*_^23^). Furthermore, according to their melting points (ZrO_2_ 2715 °C, Nb_2_O_5_ 1512 °C and Ta_2_O_5_ 1872 °C), Ta-O and Nb-O species presumably have a larger mobility on the substrate surface and within the film during growth than Zr-O, leading to well aligned BYNO nanocolumns^24^,^25^ even at high deposition rates^26^. BYTO^27^ and Ba_2_Y(Nb/Ru)O_6_^28^ have been shown to form nanocolumns in PLD-grown films, whereas BYTO forms extended nanoparticles in CSD-grown films^29^, very similar to the single perosvkites. Mixing BYNO and BYTO effectively adjusts interfacial energies and strain as well as diffusion lengths.

As shown by Ercolano *et al*.^30^, double-doping of YBCO with BYNO and BYTO leads to a further improvement of the transport properties. Furthermore, these mixed-double perovskite Ba_2_Y(Nb/Ta)O_6_-doped YBCO films (BYNTO:YBCO) seem to show a more complex *J*_*c*_ anisotropy than single-doped samples. Related structures are the so-called 2411-phases, Cu-containing mixed double perovskites, which also were shown to improve *J*_*c*_, however with less chemical stability and less tendency to form nanocolumns^31^,^32^,^33^. A deeper investigation of the material system BYNTO:YBCO is needed to provide a better understanding of its complex pinning landscape and the resulting effects on *J*_*c*_ (*H*,*T*,*θ*). This detailed study shows the influence of the microstructure of such BYNTO:YBCO thin films grown under various deposition conditions on *J*_*c*_ and *N*-value anisotropy as well as the field dependence of the pinning force density, *F*_p_(*H*), at liquid nitrogen temperatures. We explain the appearance of symmetric shoulders in *J*_*c*_ (*θ*) and *N*(*θ*) by a matching effect which does not depend on the absolute magnitude of the applied magnetic field but rather on its *c*-component.

## Results and Discussion

The growth of self-assembled nanocolumns is not only driven by interfacial energies and strain^34^,^35^,^36^ but also by diffusion of the respective atomic species, which can be controlled by temperature^37^,^38^,^39^,^40^, time (i.e. laser repetition rate)^41^,^13^ and diffusion lengths (i.e. distances between deposited material). Furthermore, secondary phases^39^ as well as growth direction (vicinality of the substrate)^42^ influence the growth kinetics of nanocolumns.

In this study, films with various laser repetition rates *f*^Dep^ between 1/2 Hz and 50 Hz were grown at fixed (optimized) temperature in order to investigate the influence of different microstructures inside the BYNTO:YBCO matrix on the transport properties.

## Microstructure

**Figure 1:**
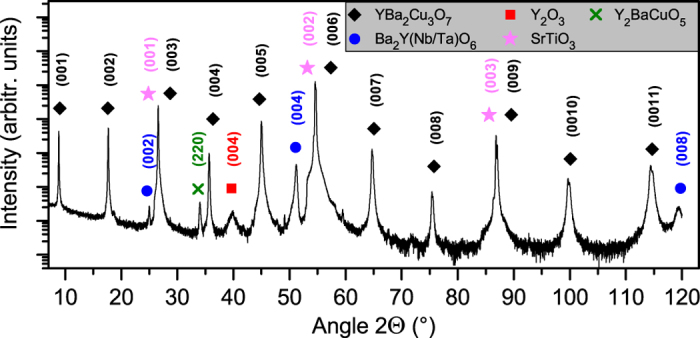
Θ-2Θ scan of the BYNTO:YBCO sample grown with *f*_Dep_ = 1 Hz. Co K_α_ was used for the measurement.

**Figure 2:**
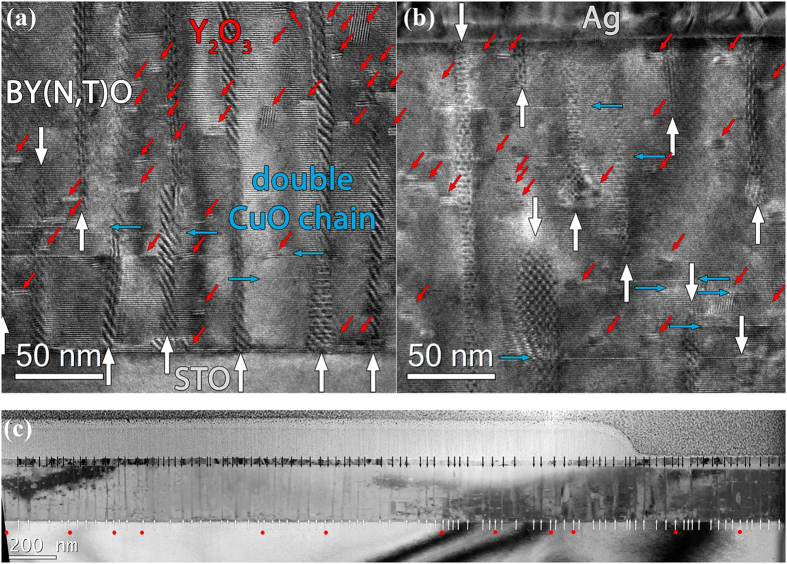
(**a,b**) HRTEM image of the 1 Hz sample at substrate-film interface (**a**) and near the film surface (**b**). Pinning centers such as BYNTO columns, Y_2_O_3_ particles and planar intergrowths are marked. (**c**) BFTEM cross section of the 1 Hz sample over an area of 3200 nm width. BYNTO columns are marked with arrows. Columns which are tilted (approx. 12% of them) are marked by red dots.

The YBCO matrix shows epitaxial growth on the STO substrates for all prepared films. As an example, Fig. 1 shows the Θ-2Θ scan for the film grown with a laser repetition rate of 1 Hz. For YBCO and BYNTO, just (00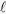) and (00 2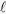) peaks are visible, respectively. Y_2_O_3_ could be found by the appearance of its (004) peak and Y_2_BaCuO_5_ by its (220) peak. BYNTO nanoparticles are aligned cube-on-cube with the YBCO for all samples. This was confirmed by selected area electron diffraction (SAED) as well as pole figure measurements of YBCO (102) and BYNTO (220), Supp. Fig. S1. The intensity of the BYNTO (00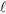) peaks and therefore the amount of biaxially incorporated BYNTO is increasing with decreasing 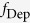 (Supp. Fig. S2). TEM cross section images of the films grown at 1 Hz (Fig. 2a,b and Supp. Fig. S4) and 5 Hz (Supp. Fig. S3) show three types of defects: BYNTO nanocolumns (horizontal), Y_2_O_3_ plates (vertical) and defects in the YBCO lattice like stacking faults (extra Y or CuO_2_ planes) or anti-phase boundaries (APB). The density and morphology of the nanoparticles depend strongly on the laser repetition rate. With increasing 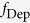 the density of the nanorods increases (1 Hz: 1 column per 29.9 nm, 5 Hz: 1 column per 14.5 nm), their diameter decreases (1 Hz: (10 ± 2) nm, 5 Hz: (8 ± 4) nm), and the density of Y_2_O_3_ plates seems to increase. Fig. 2c shows a large-area TEM cross section of 3200 nm width of the 1 Hz sample. Some of the nanorods seem to start or end within the film (red dots). However, if all columns are counted (black arrows plus red dots) an average column distance of *d* = 29.9 nm is calculated. This corresponds nicely to the value *d* = 30.5 nm obtained by plain-view TEM (Supp. Fig. S6). Therefore, we conclude that for 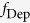 up to 5 Hz, most of the nanorods are penetrating the whole film and some are slightly inclined and cut by the lamella preparation. The components, BYNTO nanorods and Y_2_O_3_ particle, were further confirmed by EDX mapping of the contained elements (Supp. Fig. S5).

## Critical current density and Pinning force

**Figure 3:**
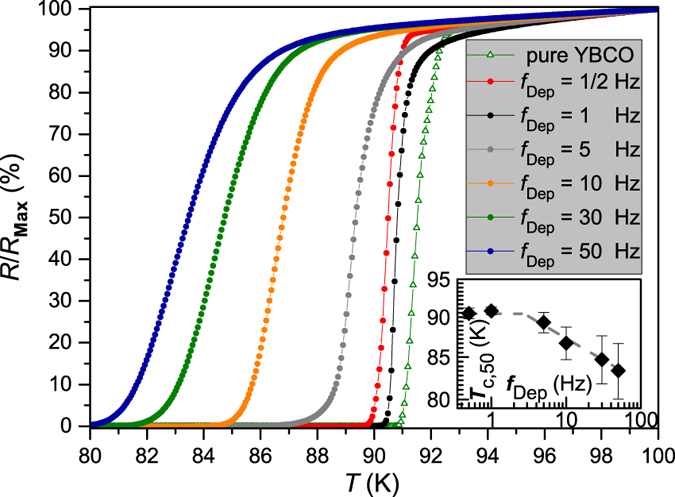
Relative resistance over applied temperature for BYNTO:YBCO films grown at laser repetition rates between 0.5 Hz and 50 Hz. Inset: Transition temperature versus laser repetition rate. Error bars indicate the transition width which was determined by taking the difference between *T*_c,__90_ and *T*_c_,_10_.

**Figure 4:**
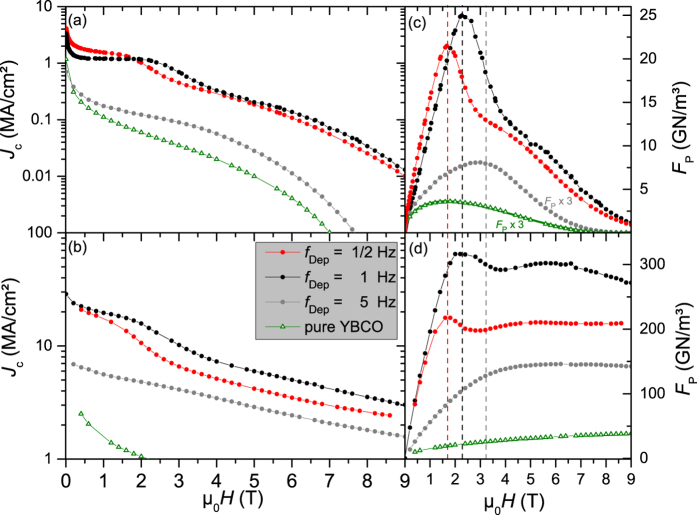
*J*_c_ versus applied magnetic field, 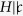, at 77 K (**a**) and 30 K (**b**) for the BYNTO:YBCO samples grown at 1/2 Hz, 1 Hz and 5 Hz as well as the corresponding *F*_P_ (μ_0_*H*) at 77 K (**c**) and 30 K (**d**). The thickness of these films is around 250 nm. Green datapoints belong to a pure YBCO film without any additions. In this case *F*_P_ (μ_0_*H*) at 77 K can be described with equation 1 (bold green line in (**c**), *p* = 0.5, *q* = 2).

Films grown at *f*_Dep_ of 1 Hz or 1/2 Hz show a transition temperature *T*_c_ of ∼90 K, which is in the experimental range of undoped samples (90…92 K). In general, *T*_c_ decreases for higher repetition rates (Fig. 3), while the transition width Δ*T*_c_ (error bars in inset of Fig. 3) is increasing by a factor of 7 (Δ*T*_c_ (1 Hz) = 0.5 K, Δ*T*_c_ (50 Hz) = 3.3 K). The reduction in *T*_c_ might be avoidable by adjusting other PLD parameters such as deposition temperature, energy density, oxygen partial pressure and target-substrate distance. Whereas the samples with 1 Hz and 1/2 Hz are similar in their current carrying capability, the samples grown at 5 Hz or higher show much smaller *J*_c_ values at 77 K (Fig. 4a and Table 1). This reduction is mainly due to the reduced *T*_c_ value because the decrease in *J*_c_ is smaller at lower temperatures such as 30 K, Fig. 4b.

The critical current density in self field *J*_c,sf_ at 77 K reaches values of 3.2 MA/cm^2^ (1 Hz) and 4.0 MA/cm^2^ (1/2 Hz) and decreases in an external field as it is expected for a high-temperature superconductors in the strong pinning limit^43^. *J*_c,sf_ values at 30 K as high as 28 MA/cm^2^ can be achieved in BYNTO:YBCO films grown with laser repetition rates of 1 Hz or below. The critical current density is above 1 MA/cm^2^ even for an external magnetic field of 9 T for all samples at this temperature (Fig. 4).The field dependence of *J*_c_ at 77 K shows an unusual behavior of samples with 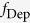 = 1 Hz and 1/2 Hz (Fig. 4a and Supp. Fig. S7). Above approx. 500 mT, *J*_c_ stays almost constant for increasing fields.

The field dependence of the pinning force density is equally unusual. For these BYNTO:YBCO thin films, it is not possible to describe 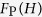 by one Dew-Hughes function^44^


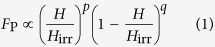


alone as it is possible for a YBCO film without any additions (bold green line in Fig. 4c). The field 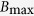 = μ_0_*H*(*F*_P,max_) at which the maximum pinning force density is reached increases with laser repetition rate (1.6 T for 1/2 Hz, 2.3 T for 1 Hz and 3.2 T for 5 Hz). Similar *J*_c_(*H*) curves have recently been reported for BaHfO_3_(BHO)-doped SmBa_2_Cu_3_O_7−*δ*_^45^. The pinning force density maximum for the 1 Hz sample (25 GN/m^3^ for the 250 nm thick film at 77 K), Fig. 4c, is among the highest values in YBCO at 77 K measured so far (e.g. 240 nm BaSnO_3_-doped: 28.3 GN/m^3 ^12, 500 nm BYNO-doped: 32.3 GN/m^3^ at 75.5 K^25^, 200 nm BaZrO_3_-doped: 21 GN/m^3^ 17). Other *RE*BCO films with slightly higher *T*_c_ values doped with BHO (370 nm GdBCO: 23.5 GN/m^3 ^46, 300 nm SmBCO: 28 GN/m^3 ^45) show similar *F*_P,max_ values at 77 K. The previously mentioned plateaus in *J*_c_(*H*) at 77 K also end at these fields 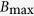. Furthermore, 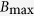 is temperature independent as can be seen by the dashed lines in Fig. 4c,d for the pinning force density at 77 K and 30 K (see also Supp. Fig. S8). Therefore, this plateau can be explained by a matching effect. Up to this field, every flux line can be pinned by an individual BYNTO column. The mean column distance of *d* = 29.9 nm, as observed in TEM for the 1 Hz sample, corresponds to a matching field 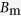 of approx. 2.3 T which corresponds quite well to the end position of the constant *J*_c_ region, respective peak position in 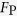
(*H*), dashed black line Fig. 4c,d (2.3 T). This value was also confirmed by a TEM plain view image where 312 columns are visible in a 0.289 μm^2^ wide area which leads to a matching field of (2.23 ± 0.14) T (Supp. Fig. S6). The same correspondence between end of *J*_c_ plateau, 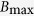 and 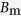 has been observed for BHO-doped SmBa_2_Cu_3_O_7−*δ*_/LAO^45^ and GdBa_2_Cu_3_O_7−*δ*_/IBAD-MgO^46^.

## The *N* value

The electric field-current density characteristics *E*(*J*) are well described by a power-law dependence, *E* ~ *J*^*N*^, over a wide electric-field range in the vicinity of *J*_c_. The *N* value has, in general, statistical and microscopic explanations. In inhomogeneous superconductors, 1/*N* is proportional to the variance in *J*_c_ as described by e.g. Warnes and Larbalestier^47^ in statistical models. The good reproducibility of *N* and *J*_c_ in our measurements however points to a microscopic explanation for *N*. It has been shown by Griessen *et al*. that in high-*T*_c_ superconductors *N* is strongly influenced if not determined by flux creep processes. Here, the flux creep rate 
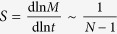
 (*M* … magnetization)^48^. Strictly, this relation only holds if *S* and *N* are measured at the same electrical field *E*. In general, *N* values determined from creep rates (equation above) are larger than from magnetization measurements^49^ and those are larger than determined from transport *E*(*J*) curves^50^ due to the negative curvature of *E*(*J*) below the glass-liquid transition. For the considerations below, this effect can be ignored, however.

**Figure 5:**
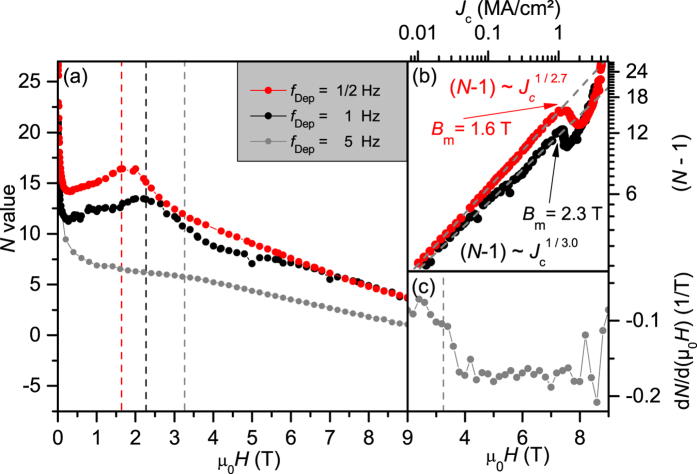
(**a**) *N*-value over field at 77 K for different samples. (**b**) *J*_c_ over *N* illustrating the reduced *N* values below 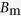. (**c**) Derivation of the *N* value for the 5 Hz sample. 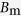
(5 Hz) is visible as step in the derivative.

For flux creep processes determined by the pinning potential 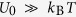 and under the assumption of a logarithmic *J*-dependence of *U*_0_^51^, 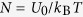 and, hence, 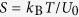 for *N* ≫ 1 (i.e. neglecting a log. time dependence term). This in turn means that *J*_c_, being determined itself by *U*_0_, scales with *N*. This is indeed found on a variety of samples in a wide range of *N* values with an empirical relation (N–1) ~ *J*_c_^1/*p*^, *p* ~ 3, Fig. 5b. The origin of this power law is beyond the scope of this paper. As seen in Fig. 5, *N* is anticorrelated to *J*_c_ in a wide magnetic field region below the matching field. The explanation for this peculiarity is a second mechanism for flux creep effects. In the presence of strongly correlated 1D or 2D pinning centers and for μ_0_*H* < *B*_m_, the flux creep rate is determined by the ability of flux half loops and double kink structures to form and move between the pinning centers, which consequently leads to an effective “hopping” of the flux lines from nanocolumn to nanocolumn. The net velocity of the flux lines and so the creep rate depends on the probability of finding a free nanocolumn in the neighborhood. Since this probability decreases with increasing magnetic field μ_0_*H* < *B*_m_, *S* decreases and *N* increases for μ_0_*H* 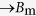. This effect is the equivalent of a Mott insulator. As observed in magnetization measurements on irradiated YBCO single crystals with columnar defects^52^, matching effects are only visible for a very narrow orientation distribution (splay) of the correlated defects, in that case of less than 4°. This, in turn, leads to an overall decreased *J*_c_ due to the increased creep rates. Larger splay values lead to lower creep rates and therefore higher *J*_c_ values but no observable matching effects. Similar reductions in creep rate due to additional defects in YBCO films have been reported recently for increased film thickness and substrate decoration^53^, and nanoparticle addition (Y211, BZO, BYTO) in films grown by metall-organic deposition (MOD)^54^,^55^. In our samples, we observe high *J*_c_ values and matching effects. This is a consequence of the intermediate Y_2_O_3_ particles and stacking faults, which hinder the half loops and double kink structures from running parallel the nanocolumns and effectively jump to the next column^13^.

## Critical current density anisotropy

**Figure 6:**
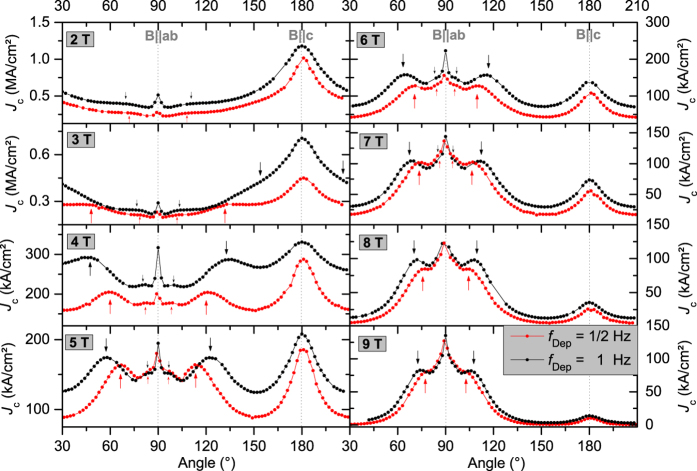
Anisotropy of the critical current density at 77 K at several fields for samples grown at low *f*_Dep_, red 0.5 Hz and black 1 Hz. Large arrows indicate the position of the main off-axis maximum, where 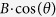 equals 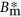 (2.0 T for the red arrows and 2.7 T for the black arrows), small arrows indicate a second off-axis maximum.

**Figure 7:**
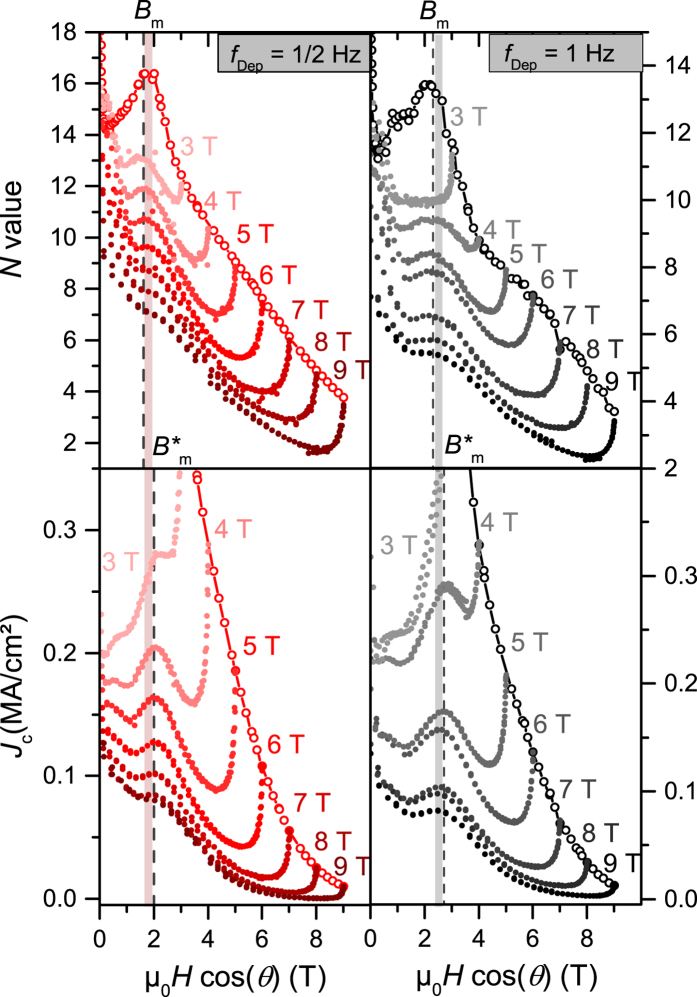
*N* value respectively *J*_c_ versus the *c*-axis component of the applied magnetic field *B*^*c*^ at 77 K. Open circles show the according data for 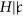 . Shaded region 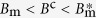 indicates anti-correlation between *N* and *J*_c_.

The anisotropy of the critical current density *J*_c_(*θ*) at 77 K shows a maximum at 90° due to the electronic anisotropy of YBCO^56^ and one at 180° due to *c*-axis correlated pinning^57^ for all BYNTO:YBCO samples. Besides these well understood features of *J*_c_(*θ*) additional maxima are visible. They appear as pairs which are oriented symmetrically around the *c*-axis peak similar to the shoulder formation seen by Ercolano *et al*.^30^ but more pronounced. The shoulder position with respect to the *c*-axis depends on the used laser repetition rate as well as the applied field, Fig. 6. Only the samples grown at a laser repetition rate of 1 Hz and 1/2 Hz will be discussed in the following. Because of the lower *J*_c_ values, the shoulders of the 5 Hz sample are barely visible. The appearance and the position of these shoulders becomes comprehensible if *N* value and *J*_c_ are plotted versus the *c*-axis component of the applied magnetic field, 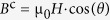, Fig. 7a,b. For both samples, the *N* value maxima (and hence minima in *S*) appear once 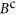 reaches the matching field 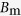 just the same as for *J*_c_ at 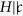. A similar effect has recently been observed by Trastoy *et al*.^58^ on YBCO films with a periodic square pattern of artificial pin holes produced by ion irradiation. They have seen strong matching effects with μ_0_*H* 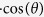 in field and angular dependence of resistivity and glass-liquid transition, concurrent with an increased mass anisotropy due to partial deoxygenation and a 2D glass-liquid transition. Our results show that this type of matching is independent of dimensionality (our samples are 3D) and periodicity (the BYNTO nanocolumns show a certain degree of density variance).

As calculated by Paulius *et al*.^59^ for correlated irradiation defects and described by e.g. Ercolano *et al*.^30^ and Jha *et al*.^60^ for nanocolumns, the vortex is not pinned by one column alone but rather by several columns through a staircase-like path for angles *θ* smaller than a certain trapping or accommodation angle^56^. The flux line segments in between the vortices are pinned by additional defects, such as Y_2_O_3_, stacking faults and antiphase boundaries, as observed in TEM. The specific position 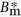, where the 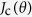 curves have their maximum in *J*_c_(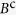) is slightly raised compared to the matching field and the maxima in the *N* value (2.0 T for 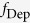 = 1/2 Hz and 2.7 T for 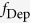 = 1 Hz, Fig. 7c,d). Apparently, the extrema in creep rate *S* and hence the *N* value are determined solely by the occupation of the *c*-axis aligned correlated defects. Therefore, they appear at the matching field 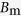. Additional intermediate defects contribute to *J*_c_ for field directions closer to *c* and lead to a further increase in *J*_c_ in a small region of 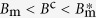. This region (shaded area in Fig. 7) shows again anti-correlation between *N* and *J*_c_ (i.e. creep rate *S* not determined by pinning potential *U*_0_). However, this configuration is different from the case 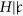, since here the anti-correlation appears above 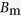, whereas it appears below 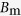 for 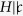 .

Besides the strongly pronounced shoulders, a second pair of shoulders can be seen for fields up to 8 T (Fig. 6, small arrows). They have a much smaller amplitude and their position is much closer to 90°. The position of those peaks does not scale with the *c*-axis component of the applied magnetic field, 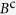. A possible scenario for those peaks might be a combined effect of fractional occupation of the correlated defects and anisotropic pinning of the flux line segments at intermediate extended defects. This has to be investigated in more detail in future studies.

## Conclusion

BYNTO-doped YBCO films were successfully grown using different laser repetition rates. Only for sufficiently long diffusion times (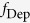 = 1 Hz or lower), very high critical current densities were achieved. All films have a rich microstructure consisting of pinning centers with different size, shape and orientation distribution. The interplay between strongly *c*-axis correlated BYNTO nanocolumns, *ab*-stacking faults, *c*-axis oriented APBs, atomic disorder and biaxially oriented but randomly distributed Y_2_O_3_ nanoparticles leads to additional new features in *F*_P_(*H*) and *J*_c_(*θ*). The critical current density shows almost constant values for magnetic fields up to several Tesla. The field where this plateau ends and where *F*_P_(*H*) has its highest value are equal to the matching field determined by TEM. A pinning force density of 25 GN/m^3^ at 77 K at the matching field of 2.3 T was achieved for the film grown at 1 Hz, which is among the highest values reported for YBCO. At 30 K, deposition rates up to 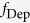 = 5 Hz lead to critical current densities of more than 1 MA/cm^2^ even in external fields of up to 9 T for 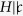.

The field dependence of the *N* value (the exponent of the *E*(*J*) curves) and its scaling behavior with the corresponding *J*_c_ value clearly show different regions of flux depinning mechanisms, being dominated by the creation of half loop and double kink structures below the matching field and by usual flux creep processes due to weaker, uncorrelated, pinning centers above the matching field.

The anisotropy of the critical current density *J*_c_(*θ*) as well as *N*(*θ*) are composed of two additional shoulder formations besides the maxima at 0° and 90° which appear at different angles if the applied magnetic field strength is changed. The existence of these shoulders can be explained by a staircase-like path of the vortices and additional pinning at intermediate defects. At a certain angle, the magnetic fields *c*-axis component μ_0_*H* ·cos *θ* reaches the matching field *B*_m_ which is accompanied by a maximum of the *N* value. Because of further contribution of intermediate defects to *J*_c_ but not to *N*, the maximum at the critical current density anisotropy 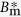 is slightly higher than the matching field *B*_m_.

Due to their ability to grow as very uniformly sized, distributed and oriented nanocolumns, Ba_2_Y(Nb/Ta)O_6_ nano-precipitates in YBa_2_Cu_3_O_7−*δ*_ thin films are not only effective artificial pinning centers for increased *J*_c_ and *F*_P_ values but also a model system to study matching effects and flux creep.

## Methods

BYNTO:YBCO films were prepared by pulsed laser deposition (PLD) using a YBCO target with 2.5 mol% BYNO and 2.5 mol% BYTO. The target was prepared by mixing the precursor oxides (barium oxide, yttrium oxide, tantalum oxide and niobium oxide) in the appropriate amount with YBCO powder and grinding the mixture in an agate mortar. The powder was pressed in pellets and sintered at 950 °C in flowing O_2_ for 24 h. A Lambda Physics LPX305i KrF excimer laser (*λ* = 248 nm, 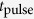 = 25 ns) was used with an energy density of 1.6 J/cm^2^ at the target surface to grow BYNTO:YBCO films of approx. 250 nm thickness on single crystalline (100)-oriented SrTiO_3_ (STO). An oxygen partial pressure of 0.4 mbar was maintained during the deposition process. The substrate temperature was set to 840 °C and checked with a HEITRONICS ceramic pyrometer. The laser repetition rate *f*_Dep_ was varied between 0.5 Hz and 50 Hz. Oxygen loading of the YBCO films took place in 400 mbar O_2_ for 1 hour at 770 °C. A silver cap layer of several nm thickness was deposited afterwards to improve the contact resistance.

Surface morphology and film thickness were analyzed by scanning electron microscopy using a JEOL JSM-6510 and an FEI Helios Nanolab 600i to cut cross sections by a focused gallium ion beam. Transmission electron microscopy (TEM) analysis was carried out on an FEI Osiris microscope, equipped with a ‘Super-X’ wide solid angle EDX detector, operated at 200 kV for bright field transmission electron microscopy (BFTEM), high angle annual dark field scanning transmission electron microscopy (HAADF STEM) and energy dispersive X-ray spectroscopy (EDX). FEI Titan ‘cubed’ electron microscope operated at 300 kV, equipped with an aberration corrector for the probe-forming lens as well as a high-brightness gun was used for high-resolution transmission electron microscopy (HRTEM). X-ray diffraction was carried out on a Bruker D8 Advance with a Co anode in a modified parallel-beam geometry for Θ-2Θ scans. Pole figures were measured at a Philips X’Pert PW3040 with a Cu anode in Bragg-Brentano geometry.

Bridges of 800 nm length and approx. 20 μm width for transport current measurements were prepared by laser cutting. Field and angular dependencies of critical current density *J*_c_ and exponent *N* of the *V*(*I*) curves were measured in a four-point assembly in magnetic fields up to 9 T with a Quantum Design physical properties measurement system (PPMS). *J*_c_ was defined by an electrical field criterion of 1 μV/cm on *V*(*I*) curves fitted as 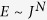 in the first decade of *E* above *E*_c_. The angular dependence of the critical current density was measured under maximum Lorentz force configuration. To correct for small heating effects, the absolute value of some of those measurements were corrected to the value measured at 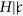 where thermal contact was better.

## Additional Information

**How to cite this article**: Opherden, L. *et al*. Large pinning forces and matching effects in YBa_2_Cu_3_O_7-δ_ thin films with Ba_2_Y(Nb/Ta)O_6_ nano-precipitates. *Sci. Rep.* **6**, 21188; doi: 10.1038/srep21188 (2016).

## Supplementary Material

Supplementary Information

## Figures and Tables

**Table 1:** Overview of the superconductivity properties of the discussed films.

*f*_Dep_	*T*_c,50_(K)	Δ*T*_c_ (K)	*J*_c,sf_ (77K) (MA/cm^2^)	*μ*_0_*H*_irr_(T)	*F*_P,max_ (77 K) (GN/m^3^)	*B*_max_ (T)	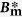 (T)
0.5	90.5	0.7	4.0	10.5	21.5	1.6	2.0
1	90.8	0.5	3.3	11.2	25.0	2.3	2.7
5	88.1	1.3	0.7	8.7	2.7	3.2	–

Δ*T*_c_ was determined by taking the difference of *T*_c,__90_ and *T*_c,__10_, 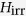 was estimated through the fit of equation (1).
