# Supplementary material for: Large pinning forces and matching effects in YBa2Cu3O7-δ thin films with Ba2Y(Nb/Ta)O6 nano-precipitates
Source: Sci Rep. 2016 Feb 18;6:21188. doi: 10.1038/srep21188 (PMC4758043; doi:10.1038/srep21188)
Supplement: Supplementary Information [file srep21188-s1.pdf]

Supplement - Large pinning forces and matching effects in $YBa_2Cu_3O_{7-\delta}$ thin films with $Ba_2Y(Nb/Ta)O_6$ nano-precipitates

Lars Opherden[1,2,3,*], Max Sieger[1], Patrick Pahlke[1,2], Ruben Hühne[1], Ludwig Schultz[1,2], Alexander Meledin[4], Gustaaf Van Tendeloo[4], Rainer Nast[5], Bernhard Holzapfel[5], Marco Bianchetti[6], Judith L. MacManus-Driscoll[6], and Jens Hänisch[1,5,*]

[1]IFW Dresden, Institute for Metallic Materials, P.O. Box 270116, 01171 Dresden, Germany
[2]TU Dresden, Institute for Solid-State Physics, 01062 Dresden, Germany
[3]Dresden High Magnetic Field Laboratory, Helmholtz-Zentrum Dresden-Rossendorf, 01314 Dresden, Germany
[4]University of Antwerp, EMAT Research Group, Groenenborgerlaan 171, 2020 Antwerp, Belgium
[5]KIT, Institute for Technical Physics, Hermann-von-Helmholtz-Platz 1, 76344 Eggenstein-Leopoldshafen, Germany
[6]University of Cambridge, Department of Materials Science and Metallurgy, 27 Charles Babbage Rd., Cambridge, CB3 0FS, U.K.
[*]l.opherden@hzdr.de, jens.haenisch@kit.edu

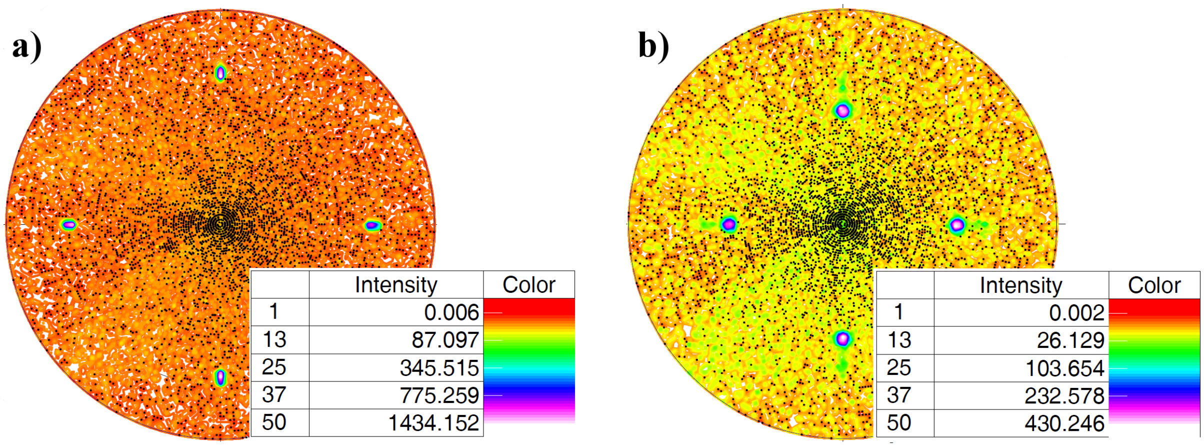

Figure S1. Pole figures of the 1 Hz film for a) YBCO (102) and b) BYNTO (220). On the polar axis shows Ψ between 0° and 90°, the azimuth shows Φ between 0° and 360°. BYNTO is aligned cube-on-cube with the YBCO.

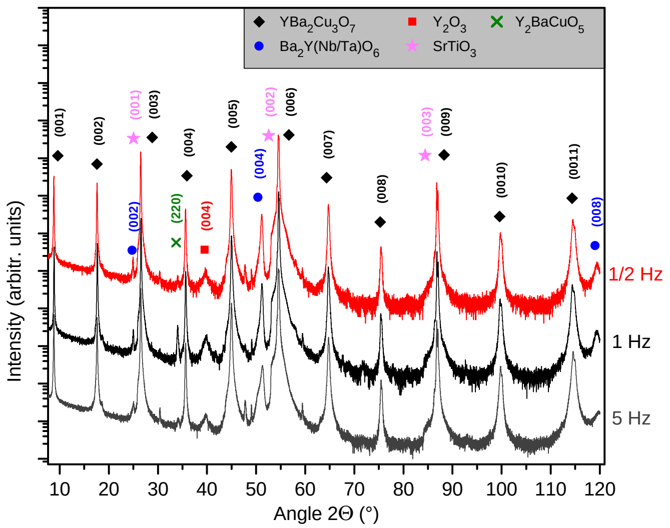

Figure S2. Θ-2Θ scan of the BYNTO:YBCO samples grown with different f_{Dep} in comparison. Co K_α was used for the measurement. While width and height of the YBCO peaks are the same for all three discussed deposition frequencies the amount of oriented BYNTO is increased with decreasing f_{Dep}. The Y_2O_3 peak intensity is lowered for the film grown at 5 Hz. Y_2BaCuO_5 appears also in different orientations if f_{Dep} of 5 Hz or 1/2 Hz was used.

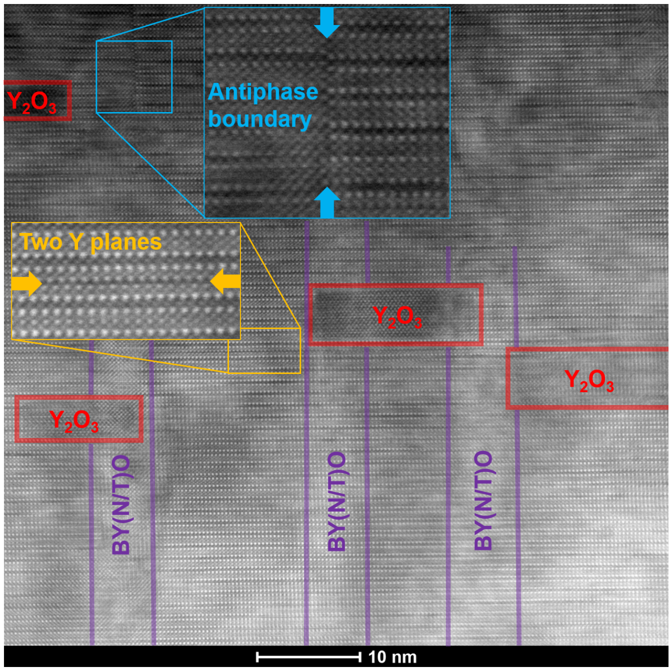

Figure S3. HAADF STEM image of the 5 Hz sample. Different defect types are marked.

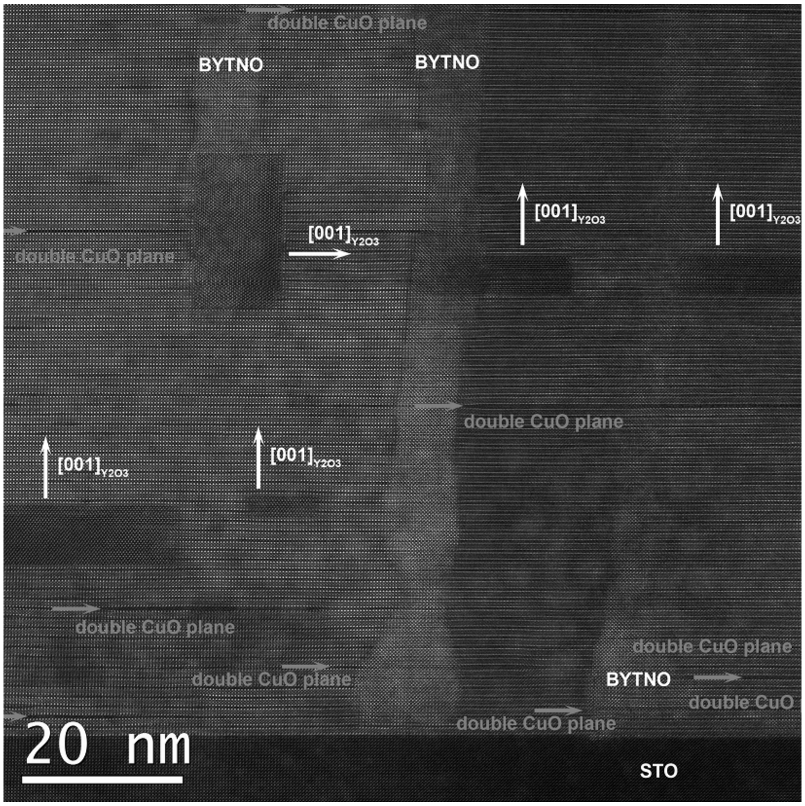

Figure S4. The rich microstructure of the 1 Hz sample is clearly visible in HRTEM images. The nucleation of the BYNTO rods seems to be not directly at the substrate interface. Mostly, the growth starts after a few YBCO unit cells. Occasionally, the growth starts somewhere in the middle of the film. In that case, a disturbed region is seen in the YBCO matrix below the column.

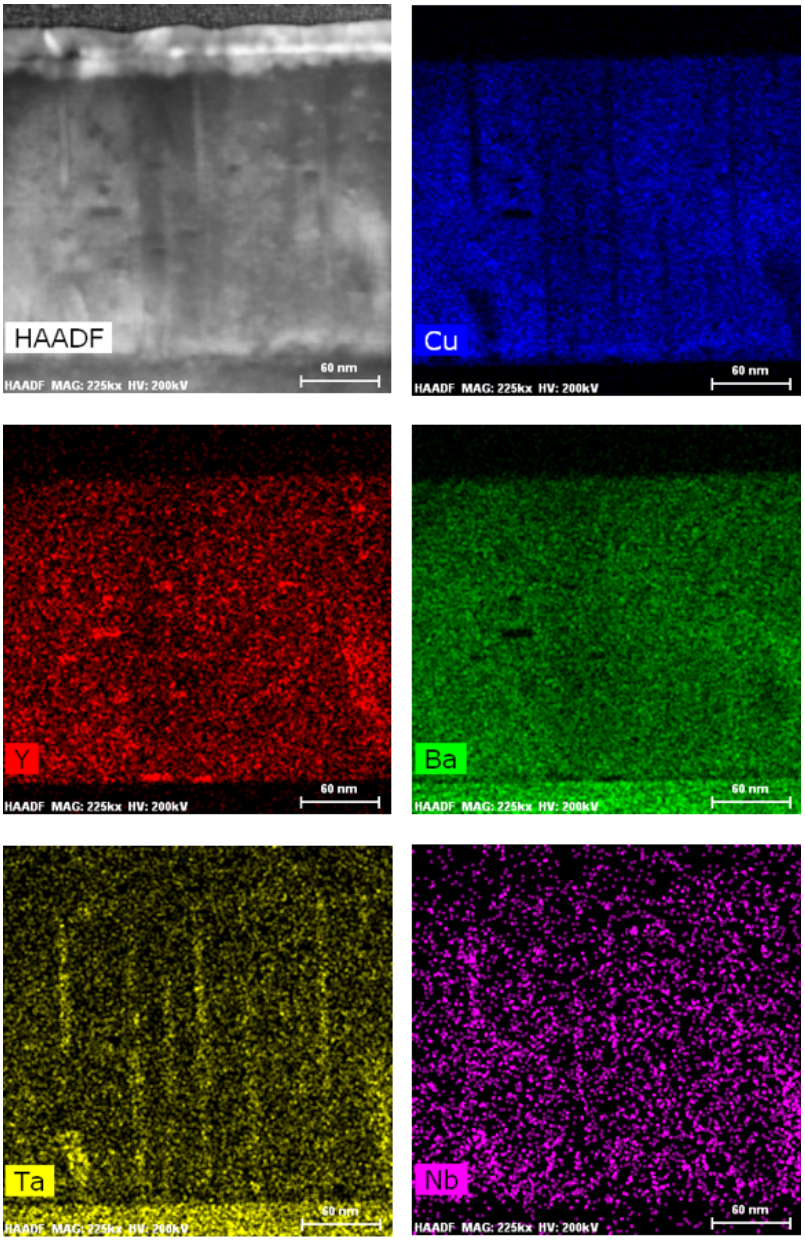

Figure S5. EDX maps of several elements.Y_2O_3 particles (middle) and BYNTO columns (down) are clearly visible.

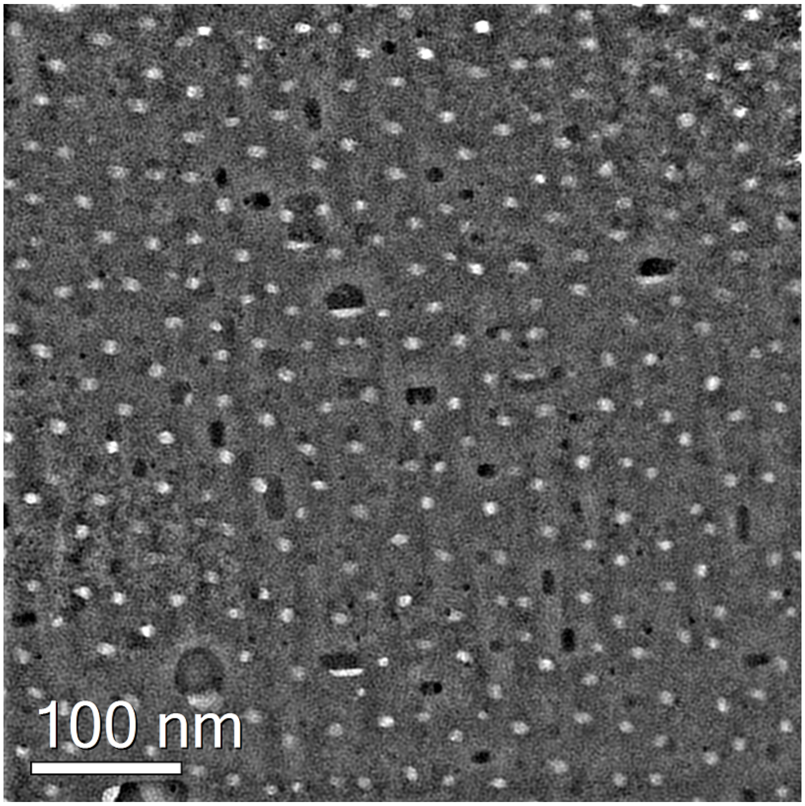

Figure S6. TEM plain view image of the 1 Hz sample. 312 columns are visible in a 0.289 μm^2 wide area which leads to a matching field of 2.23 T ± 0.14 T. This value and its error was estimated by cutting the image in 4 parts of the same size and calculating the matching field in each of them. This value has a level of reliability of 3 σ.

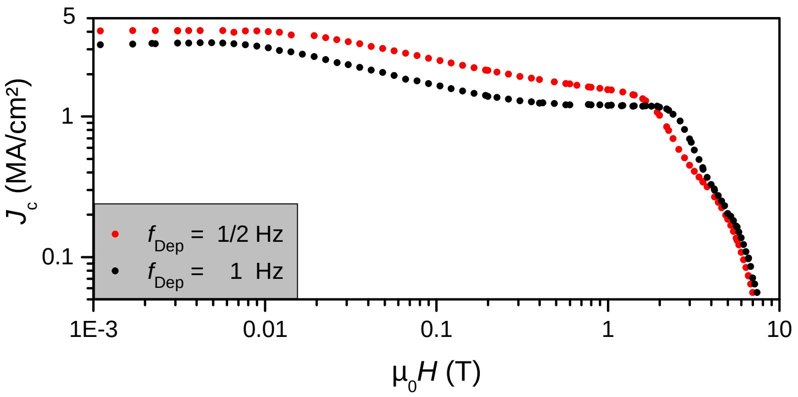

Figure S7. J_c versus applied field at 77 K, $H \| c$ for the BYNTO:YBCO samples grown at low laser repetition rates.

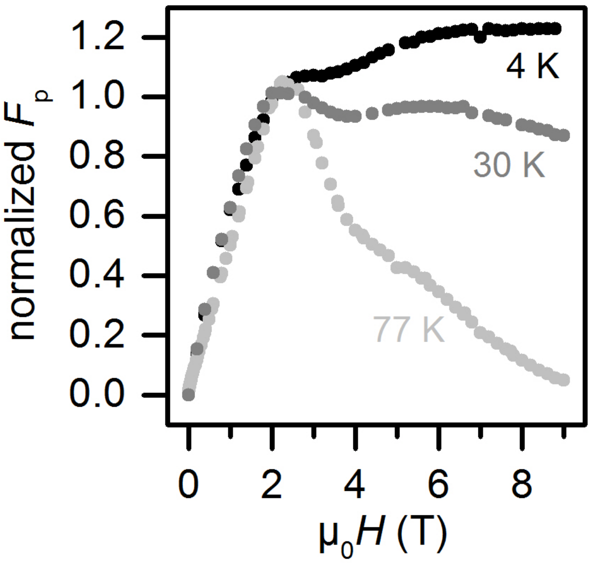

Figure S8. Normalized pinning force density F_p versus applied field of the 1 Hz sample measured for different temperatures. It is clearly visible that the maximum B_{max} appears at the matching field independently of the temperature.
